# Individualized treatment recommendations for patients with locally advanced head and neck squamous cell carcinoma utilizing deep learning

**DOI:** 10.3389/fmed.2024.1478842

**Published:** 2025-01-06

**Authors:** Linmei Zhang, Enzhao Zhu, Jiaying Shi, Xiao Wu, Shaokang Cao, Sining Huang, Zisheng Ai, Jiansheng Su

**Affiliations:** ^1^Shanghai Engineering Research Center of Tooth Restoration and Regeneration, Tongji Research Institute of Stomatology, Department of Prosthodontics, Shanghai Tongji Stomatological Hospital, Dental School, Tongji University, Shanghai, China; ^2^School of Medicine, Tongji University, Shanghai, China; ^3^Shanghai Engineering Research Center of Tooth Restoration and Regeneration, Tongji Research Institute of Stomatology, Department of Periodontics, Shanghai Tongji Stomatological Hospital, Dental School, Tongji University, Shanghai, China; ^4^Shanghai Engineering Research Center of Tooth Restoration and Regeneration, Tongji Research Institute of Stomatology, Department of Oral and Maxillofacial Surgery, Shanghai Tongji Stomatological Hospital, Dental School, Tongji University, Shanghai, China; ^5^Shanghai Engineering Research Center of Tooth Restoration and Regeneration, Tongji Research Institute of Stomatology, Department of Oral Implantology, Shanghai Tongji Stomatological Hospital, Dental School, Tongji University, Shanghai, China; ^6^Department of Medical Statistics, School of Medicine, Tongji University, Shanghai, China

**Keywords:** head and neck squamous cell carcinoma, chemoradiotherapy, deep learning, causal inference, precise medicine

## Abstract

**Background:**

The conventional treatment for locally advanced head and neck squamous cell carcinoma (LA-HNSCC) is surgery; however, the efficacy of definitive chemoradiotherapy (CRT) remains controversial.

**Objective:**

This study aimed to evaluate the ability of deep learning (DL) models to identify patients with LA-HNSCC who can achieve organ preservation through definitive CRT and provide individualized adjuvant treatment recommendations for patients who are better suited for surgery.

**Methods:**

Five models were developed for treatment recommendations. Their performance was assessed by comparing the difference in overall survival rates between patients whose actual treatments aligned with the model recommendations and those whose treatments did not. Inverse probability treatment weighting (IPTW) was employed to reduce bias. The effect of the characteristics on treatment plan selection was quantified through causal inference.

**Results:**

A total of 7,376 patients with LA-HNSCC were enrolled. Balanced Individual Treatment Effect for Survival data (BITES) demonstrated superior performance in both the CRT recommendation (IPTW-adjusted hazard ratio (HR): 0.84, 95% confidence interval (CI), 0.72–0.98) and the adjuvant therapy recommendation (IPTW-adjusted HR: 0.77, 95% CI, 0.61–0.85), outperforming other models and the National Comprehensive Cancer Network guidelines (IPTW-adjusted HR: 0.87, 95% CI, 0.73–0.96).

**Conclusion:**

BITES can identify the most suitable treatment option for an individual patient from the three most common treatment options. DL models facilitate the establishment of a valid and reliable treatment recommendation system supported by quantitative evidence.

## Introduction

Head and neck squamous cell carcinoma (HNSCC) is one of the most prevalent cancers worldwide (1), often diagnosed at an advanced stage due to the lack of effective early screening strategies (2).

Conventional treatment typically involves surgery followed by radiotherapy (RT) (3). While adjuvant chemoradiotherapy (CRT) has been shown to enhance progression-free survival by sensitizing tumors to RT under certain conditions (4), its use is controversial due to potential toxicity and complications (5).

Furthermore, the trauma and dysfunction associated with surgery have prompted interest in definitive CRT for organ preservation (6). Studies have indicated that CRT may improve outcomes in patients with non-T4 disease and high nodal burden compared to surgery, which, conversely, may benefit T4 patients (7). The response of patients to the same treatment is influenced by many underlying clinical features (8), suggesting significant treatment heterogeneity.

Given the challenges and costs associated with conducting randomized clinical trials, there is a growing demand for innovative survival analysis methods to address this heterogeneity (8). Deep learning (DL) has proven to be more accurate than traditional statistical analysis (9) and has demonstrated the potential to provide individualized recommendations based on calculated risk (10).

This study aimed to assess DL's capability to provide individualized treatment recommendations, identifying patients who might benefit from organ preservation through CRT and tailoring adjuvant treatment for those better suited for surgical interventions.

## Methods

### Study design and data source

This was a population-based retrospective cohort study designed to provide personalized treatment recommendations for locally advanced HNSCC (LA-HNSCC) patients using DL models. The evaluation of the treatment options was categorized into two phases, with phase one individualizing treatment recommendations between CRT and surgery plus CRT/RT and phase two individualizing treatment recommendations between surgery plus CRT and surgery plus RT.

The population for this study was sourced from the Surveillance, Epidemiology, and End Results (SEER) 18 database, which represents approximately 27.8% of the U.S. population (11). This study followed the Strengthening the Reporting of Observational Studies in Epidemiology guidelines (12).

### Study population and eligibility criteria

Patients with HNSCC originating from four anatomical sites (such as the oral cavity, sinonasal cavity, pharynx, and larynx), diagnosed as stage III to IVa from 1 January 2004 to 31 December 2015, and treated with definitive CRT or radical resection plus postoperative RT/CRT were included in this study. Nasopharyngeal and salivary gland carcinomas were not included due to differences in pathology and treatment.

Ethnicity (13), sex (13), marital status (14), age (15), histological grade (16), laterality (17), primary tumor site (18), TNM stage (3), tumor size (3), number of lymph nodes (19), number of positive lymph nodes (20), and lymph node surgery (21) were included as variables affecting efficacy because they are known to play critical roles in predicting prognosis and guiding treatment decisions in HNSCC. OS was used to measure the efficacy of each treatment regimen.

**Figure 1:**
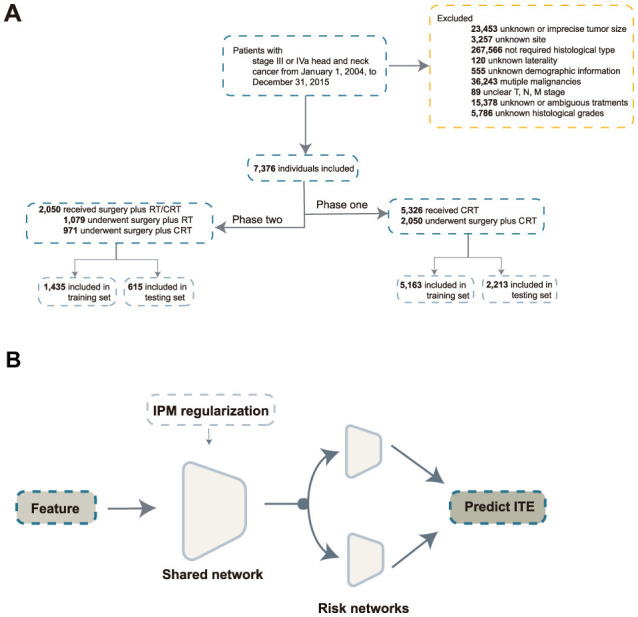
Inclusion process and model architecture. **(A)** Inclusion process; **(B)** architecture of the balanced individual treatment effect for survival data. RT, radiation; CRT, chemoradiation; IPM, integral probability metrics; ITE, individual treatment effect.

Clinical cases were excluded if they met the following criteria: (1) unknown or ambiguous demographic information; (2) unknown histologic grades or tumor type; (3) unknown tumor location or size; (4) unknown TNM stage; (5) unknown treatment modality; (6) stage I, II, or IVb; (7) unknown laterality; (8) incomplete follow-up; (9) multiple malignancies; and (10) metastatic tumors. The cohort selection is illustrated in Figure 1A.

TNM stage was determined in accordance with the 7th American Joint Committee on Cancer staging manual. Patients who were alive as of 31st December 2020 were censored. Therefore, the follow-up period ranged from 5 to 16 years.

### Algorithms

The individual treatment effect (ITE) reflects the difference in survival outcomes between two potential intervention scenarios. The T-learner is a common type of model used for inferring the ITE, which adopts two models to estimate the ITE as *ITE* = μ_1_(*x*)− μ_0_(*x*), where μ_0_ and μ_1_ denote the models trained on the corresponding treatment groups (22). The T-learner excludes some confounding artifacts; however, it can still be affected by inconsistent predictive performance of models (23) and biased treatment allocation (24).

With the development of DL, more methods have been proposed to estimate the unbiased ITE. Balanced Individual Treatment Effect for Survival data (BITES) (24) addresses this issue through representation-based causal inference. BITES has a shared network and two risk networks. In the shared network, integral probability metrics are used to maximize the p-Wasserstein distance of different treatment arms. The risk networks calculate the ITE in the form of a T-learner. The architecture of BITES is illustrated in Figure 1B.

Cox Mixtures with Heterogeneous Effects (CMHE) (25) uses a latent variable approach to model heterogeneous treatment effects by assuming that an individual can belong to one of the latent clusters with distinct response characteristics.

### Calculation of the individual treatment effect

For censored data, the models output log hazard ratios; however, these cannot be used directly because the baseline hazards of different treatment groups also reflect crucial prognostic information.

Here, we defined the potential outcome with a good clinical interpretation as the area under the individual survival curve for an individual within a specific period (5 years), called the restricted survival time (RST). The formula was described as $ITE_{RST}\left(X;t\right)=\underset{x\in X}{\sum }\left[\overset{t}{\underset{0}{\int }}{Ŝ}_{1}\left(t|x\right)dt-\overset{t}{\underset{0}{\int }}{Ŝ}_{0}\left(t|x\right)dt\right]$, where *t* indicated the preset time horizon and Ŝ_0_(*t*∣*x*) and Ŝ_1_(*t*∣*x*) were the predicted survival distributions for an individual under different treatments. It can be simply interpreted as the additional amount of time a patient survived within 5 years when receiving treatment 1 compared with receiving treatment 0.

### Model development, validation, and treatment recommendation

We trained and compared five models, including BITES, CMHE, DeepSurv (26), the Cox proportional hazards (CPH) model, and random survival forest (RSF). These models, divided into deep learning models (BITES, CMHE, and DeepSurv) and traditional machine learning models (CPH and RSF), all employed the same ITE calculation method. The deep learning models were chosen for their ability to capture complex non-linear relationships, while the traditional models were used as benchmarks for performance comparison.

All patients were randomly allocated to a training set comprising 70% of the samples used for training the models and a testing set comprising 30% of the samples to evaluate the model performance and recommendation effect. During the training period, we used five-fold cross-validation to tune the model hyperparameters. Each time, the model was trained on four-fifths of the training set and validated on the remaining one-fifth. The training process was automatically terminated if the validation loss did not decrease after 1,000 iterations. Hyperparameter tuning was conducted using grid search to explore the predefined ranges of key parameters. These parameters included learning rate, mini-batch size, the percentage of dropout, number of layers, number of nodes in the multilayer perceptron, strength of the regularization method, number of trees, and tree depth, depending on the model. The optimal hyperparameters were selected based on the validation loss.

To evaluate the models' treatment recommendation effect, the patients were divided into the recommended (Consis.) and anti-recommended (Inconsis.) groups, based on whether the actual treatment they received was consistent with the model recommendations. We calculated several indicators between the Consis. and Inconsis. groups to quantify the survival advantages of the following models' recommendations: multivariate hazard ratio (HR), 5-year absolute risk reduction (ARR), and the difference in restricted mean survival time (DRMST) over five years. Considering the potential imbalance of the baseline features between the Consis. and Inconsis. groups, inverse probability treatment weighting (IPTW) was used to reduce selection bias.

### Model interpretation

The model interpretation was twofold: (1) the importance of the features for the overall output and (2) the impact of the features on the treatment recommendations.

SHapley Additive exPlanations (SHAP) is a widely used local interpretation method from game theory that explains the extent to which each variable affects the model output with respect to the baseline average. In this study, we employed SurvSHAP(t) (27), a time-dependent SHAP analysis, to explain the output of the best model.

We calculated the probability that a patient with a certain characteristic is recommended for a specific treatment minus the probability that a patient without that characteristic is recommended for the same treatment. This difference is called the probability difference (PD), which is similar to the calculation of risk difference. Based on the PD, the impact of features on treatment recommendations can be quantified. We also used IPTW to exclude the influence of other characteristics, thereby obtaining the independent impact.

### Statistical analysis

The models were built using Python 3.8 with the packages Pytorch 2.0 and Scikit-survival 0.19.0. Statistical analyses were performed using R 4.1.38. Continuous variables were expressed as medians and interquartile ranges (IQRs), and categorical variables were expressed as numbers and percentages (%). The log-rank test was used to compare the Kaplan–Meier (KM) curves.

## Results

### Patients

A total of 7,376 patients with locally advanced HNSCC were enrolled, with a median follow-up of 58 (IQR: 16–102) months, including 3,613 (49.0%) patients with oral cavity cancer, 2,041 (27.7%) patients with pharyngeal cancer, 59 (0.8%) patients with sinonasal cavity cancer, and 1,663 (22.5%) patients with laryngeal cancer. Of these, 5,326 patients were treated with CRT and 2,050 patients were treated with surgery. Adjuvant RT was administered to 1,079 of the patients who underwent surgery, and adjuvant CRT was administered to an additional 971 patients. The overall mortality rate was 61.6% [95% confidence interval (CI): 60.5%−62.8%]. The detailed baseline demographic and clinical characteristics of the included patients are presented in Table 1.

**Table 1:** Patients.

	**Concurrent chemoradiation (*n* = 5,326)**	**Surgery and postoperative radiation (*n* = 1,079)**	**Surgery and postoperative chemoradiation (*n* = 971)**
Age, median (IQR), y	60.0 (53.0–67.0)	61.0 (54.0–70.0)	59.0 (53.0–60.0)
Tumor size, median (IQR), mm	32.0 (24.0–44.0)	35.0 (25.0–45.0)	38.0 (27.0–50.0)
Married	2,911 (54.7)	540 (50.1)	494 (50.9)
Ethnicity–White	4,496 (84.4)	859 (79.6)	770 (79.3)
Male	4,410 (82.8)	787 (72.9)	742 (76.4)
**Grade**			
I	333 (6.3)	111 (10.3)	86 (8.9)
II	2,565 (48.2)	670 (62.1)	532 (54.8)
III	2,380 (44.7)	295 (27.3)	350 (36.0)
IV	48 (0.9)	3 (0.3)	3 (0.3)
**Laterality**			
Left	960 (18.0)	84 (7.8)	92 (9.5)
Right	1,031 (19.4)	95 (8.8)	90 (9.3)
Not paired	3,335 (62.6)	90 (83.4)	789 (81.3)
**Oral cavity**			
Lip	1 (0.0)	17 (1.6)	6 (0.6)
Base of tongue	1,832 (34.4)	48 (4.4)	76 (7.8)
Other parts of tongue	230 (4.3)	105 (9.7)	132 (13.6)
Gum	23 (0.4)	144 (13.3)	95 (9.8)
Palate	115 (2.2)	32 (3.0)	30 (3.1)
Floor of mouth	82 (1.5)	166 (15.4)	135 (13.9)
Mouth	81 (1.5)	131 (12.1)	132 (13.6)
**Pharynx**			
Tonsil	1,432 (26.7)	7 (0.6)	14 (1.4)
Oropharynx	106 (2.0)	1 (0.1)	1 (0.1)
Pyriform	270 (5.1)	3 (0.3)	0 (0.0)
Hypopharynx	207 (3.9)	3 (0.3)	6 (0.6)
Paranasal sinus	30 (0.6)	17 (1.6)	12 (1.2)
Larynx	926 (17.4)	405 (37.5)	332 (34.2)
**Stage**			
III	1,501 (28.2)	289 (26.8)	153 (15.8)
IVa	3,825 (71.8)	790 (73.2)	818 (84.2)
**T stage**			
T1	706 (13.3)	77 (7.1)	69 (7.1)
T1NOS	4 (0.1)	1 (0.1)	0 (0.0)
T1a	2 (0.0)	0 (0.0)	0 (0.0)
T1b	1 (0.0)	1 (0.1)	0 (0.0)
T2	2,071 (38.9)	163 (15.1)	165 (17.0)
T3	1,422 (26.7)	262 (24.3)	194 (20.0)
T4a	1,120 (21.0)	575 (53.3)	543 (55.9)
**N stage**			
N0	669 (12.6)	451 (41.8)	195 (20.1)
N1	1,320 (24.8)	257 (23.8)	205 (21.1)
N2NOS	112 (2.1)	7 (0.6)	8 (0.8)
N2a	398 (7.5)	32 (3.0)	38 (3.9)
N2b	1,685 (31.6)	232 (21.5)	331 (34.1)
N2c	1,142 (21.4)	100 (9.3)	194 (20.0)
Follow-up, median (IQR), month	64.0 (17.0–107.0)	41.0 (14.0–89.0)	33.0 (13.0–84.0)

### Performance

All evaluations of the model were performed on the testing set, which included 2,213 patients for the phase one and 651 patients for phase two recommendations. The detailed model performance is presented in Table 2.

**Table 2:** Performance.

**Model**	**HR**	**IPTW-adjusted HR**	**5-year DRMST (month)**	**IPTW-adjusted 5–year DRMST (month)**	**5-year ARR (%)**	**IPTW-adjusted 5-year ARR (%)**	**IBSa**	**IBSb**
**Chemoradiation vs. surgery plus radiation/chemoradiation**								
BITES	0.92 (0.81–1.04)	**0.84 (0.72–0.98)**	6.71 (4.75–8.67)	**10.40 (8.33–12.75)**	**16.90 (12.50–21.20)**	**14.80 (10.60–19.10)**	0.21 (0.21–0.22)	0.19 (0.18–0.20)
CMHE	0.77 (0.67–0.89)	Reference	−0.23 (−2.16–1.71)	4.25 (2.20–6.36)	−2.71 (−7.04–1.63)	−1.78 (−5.98–2.42)	0.20 (0.19–0.20)	0.22 (0.22–0.23)
DeepSurv	0.77 (0.67–0.89)	Reference	−0.23 (−2.16–1.71)	Reference	−2.64 (−6.97–1.69)	−1.79 (−5.99–2.42)	0.37 (0.35–0.39)	0.29 (0.27–0.32)
RSF	0.82 (0.73–0.92)	0.85 (0.75–0.96)	**7.37 (5.48–9.25)**	9.70 (7.65–12.49)	13.90 (9.78–18.10)	10.20 (6.11–14.30)	0.17 (0.17–0.18)	0.18 (0.16–0.19)
CPH	**0.76 (0.67–0.86)**	0.98 (0.78–1.24)	3.74 (1.88–5.61)	4.84 (2.72–6.25)	7.52 (3.34–11.70)	6.93 (2.89–11.00)	**0.17 (0.16–0.18)**	**0.17 (0.16–0.18)**
NCCN	0.88 (0.73–1.06)	0.87 (0.73–0.96)	−4.12 (−6.31–−1.92)	−4.37 (−6.40-−2.12)	−9.29 (−14.20–−4.43)	−8.34 (−13.00–−3.65)	..	..
**Model**	**HR**	**IPTW–adjusted HR**	**5–year DRMST (month)**	**IPTW–adjusted 5–year DRMST (month)**	**5–year ARR (%)**	**IPTW–adjusted 5–year ARR (%)**	**IBSc**	**IBSd**
**Surgery plus radiation vs. surgery plus chemoradiation**								
BITES	0.87 (0.72–1.06)	**0.77 (0.61–0.85)**	**4.59 (1.18–8.01)**	**4.65 (1.32–7.73)**	**11.10 (3.58–18.60)**	**10.50 (3.16–17.90)**	0.22 (0.21–0.23)	0.20 (0.18–0.22)
CMHE	**0.82 (0.66–1.03)**	0.83 (0.65–1.07)	3.56 (0.14–6.98)	3.55 (0.29–7.36)	4.60 (−2.96–12.20)	4.66 (−2.75–12.10)	0.23 (0.22–0.23)	0.22 (0.21–0.23)
DeepSurv	0.93 (0.76–1.15)	0.94 (0.76–1.17)	−1.26 (−4.69–2.18)	−1.22 (−4.80–2.02)	−1.51 (−9.09–6.07)	−1.31 (−8.75–6.14)	0.33 (0.30–0.37)	0.45 (0.40–0.48)
RSF	0.86 (0.71–1.04)	0.90 (0.73–1.10)	2.55 (−0.91–6.00)	2.59 (−0.73–5.94)	6.88 (−7.23–14.50)	6.37 (−1.10–13.80)	0.17 (0.16–0.19)	0.18 (0.17–0.20)
CPH	0.84 (0.67–1.05)	0.79 (0.61–1.02)	3.66 (0.13–7.18)	3.61 (0.04–7.22)	7.93 (0.19–15.70)	7.39 (−0.23–15.00)	**0.17 (0.15–0.18)**	**0.18 (0.16–0.21)**

IPTW, inverse probability weighting; HR, multivariate hazards ratio; DRMST, the difference in restricted mean survival time; ARR, absolute risk reduction; IBSa, integrated Brier score in chemoradiation group; IBSb, integrated Brier score in surgery plus radiation/chemoradiation group; IBSc, integrated Brier score in surgery plus radiation group; IBSd, integrated Brier score in surgery plus chemoradiation group; BITES, Balanced Individual Treatment Effect for Survival data; CMHE, Cox Mixtures with Heterogeneous Effects; CPH, Cox proportional hazards model; RSF, random survival forest; NCCN, National comprehensive cancer network guideline; Reference, statistical model did not fit.

The bold font indicates that the model performed best in this metric.

According to the NCCN guidelines, surgery is recommended for patients with the following location characteristics and TNM stages: oral cavity cancer patients with T3 and N0, T1-3 and N 1-3, or T4a and N0-3; laryngeal cancer patients with T4a and N-3; ethmoid sinus patients with T3–4a; and maxillary sinus patients with T3–4 and N0 or T1–4a and N+.

The integrated Brier score (IBS) was used to measure the discrimination of the models. The CPH model was observed to have the best discrimination in both phase one (IBS in the CRT group (IBS^a^): 0.17, 95% CI, 0.16–0.18; IBS in the surgery plus RT/CRT group (IBS^b^): 0.17, 95% CI, 0.16–0.18) and phase two recommendations (IBS in the surgery plus RT group (IBS^c^): 0.17, 95% CI, 0.15–0.18; IBS in the surgery plus CRT group (IBS^d^): 0.18, 95% CI, 0.16–0.21), followed by the RSF model (IBS^a^: 0.17, 95% CI, 0.17–0.18; IBS^b^: 0.18, 95% CI, 0.16–0.19; IBS^c^: 0.17, 95% CI, 0.16–0.19; IBS^d^: 0.18, 95% CI, 0.17–0.20).

The metric of interest lies in how much survival advantage can be gained by following model recommendations. IPTW was used to adjust for tumor size, tumor locations, laterality, TNM stages, demographic features, and actual treatments. We set the metrics that determined the performance of the model to those corrected with IPTW, as they were largely unaffected by other factors as well as the actual treatment proportions.

In the phase one recommendation, BITES performed the best (HR: 0.92, 95% CI, 0.81–1.04; IPTW-adjusted HR (HR^e^): 0.84, 95% CI, 0.72–0.98; DRMST: 6.71, 95% CI, 4.75–8.67; IPTW-adjusted DRMST (DRMST^e^): 10.40, 95% CI, 8.33–12.75; ARR: 16.90, 95% CI, 12.50–21.20; IPTW-adjusted ARR (ARR^e^): 14.80, 95% CI, 10.60–19.10). The NCCN Clinical Practice Guidelines in Oncology (NCCN Guidelines) were also compared with the models. The patients whose actual treatment was consistent with the NCCN guidelines were compared with those whose treatment was inconsistent. As the NCCN has no prioritized treatment guidelines for pharyngeal cancers, these patients were excluded from this calculation. No significant differences were observed in the results of the NCCN guideline recommendations (HR^e^: 0.87, 95% CI, 0.73–0.96; DRMST^e^: −4.37, 95% CI, −6.40-−2.12; ARR^e^: −8.34, 95% CI, −13.00-−3.65).

For the phase two recommendation, the BITES model was noteworthy (HR: 0.87, 95% CI, 0.72–1.06; HR^e^: 0.77, 95% CI, 0.61–0.85; DRMST: 4.59, 95% CI, 1.18–8.01; DRMST^e^: 4.65, 95% CI, 1.32–7.73; ARR: 11.10, 95% CI, 3.58–18.60; ARR^e^: 10.50, 95% CI, 3.16–17.90), outperforming all other models.

**Figure 2:**
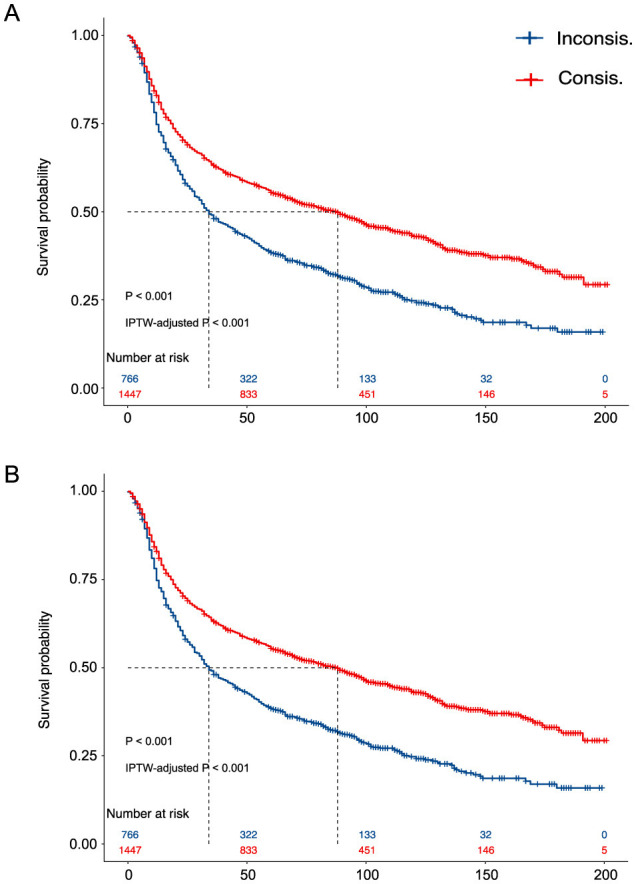
The Kaplan–Meier curves of the Consis. Group vs. the Inconsis. **(A)** The Kaplan–Meier curves of the phase one recommendation; **(B)** The Kaplan–Meier curves of the phase two recommendation. P, the *p*-value of the log-rank test; IPTW, inverse probability treatment weighting.

We present the KM curves of the Consis. vs. Inconsis. groups for the phase one and phase two recommendations in Figures 2A, B, respectively. Better OS in the Consis. group was observed for both phase one (*P* of the log-rank test < 0.001; *P* of the IPTW-adjusted log-rank test < 0.001) and phase two (*P* of the log-rank test < 0.001; *P* of the IPTW-adjusted log-rank test < 0.001) recommendations.

**Figure 3:**
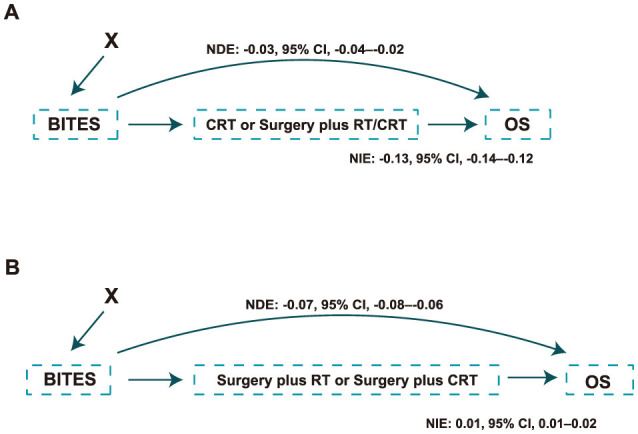
Causal path of the protection effect of the model recommendation. **(A)** Causal path of the protection effect in the phase one recommendation; **(B)** Causal path of the protection effect in the phase two recommendation. NDE, natural direct effect; NIE, natural indirect effect; BITES, Balanced Individual Treatment Effect for Survival data; OS, overall survival; RT, radiation; CRT, chemoradiation.

Whether the protective effect of BITES was due to an imbalance in the treatment proportions in the two groups was also of interest. Thus, we treated surgery plus RT/CRT as a mediator and adjusted for all baseline features to calculate the natural direct effect (NDE) and natural indirect effect, which are presented in Figure 3A. Similarly, surgery plus CRT was treated as a mediator in the evaluation of the phase two recommendation (Figure 3B). The NDE measured the direct effect of BITES recommendation on mortality reduction, excluding the effect of the actual treatment. These values are presented as the slope of a linear regression. Both phase one (NDE: −0.03, 95% CI, −0.04–−0.02) and phase two (NDE: −0.07, 95% CI, −0.08–−0.06) recommendations had a direct effect on overall mortality reduction.

We also assessed the protective effect of BITES on various causes of death, as presented in Supplementary Table S1. As competing risks were considered, when a particular cause of death was tested, other deaths were treated as competing risks. The HR^e^ with the competing risks was calculated using a marginal structural cause-specific Cox proportional hazards model (MSM) (28). For the phase one recommendation, the patients who followed the model recommendation had a lower death rate from HNSCC (HR^e^: 0.84, 95% CI, 0.69–0.94), cardiovascular diseases (HR^e^: 0.66, 95% CI, 0.45–0.96), and adverse effects (HR^e^: 0.68, 95% CI, 0.38–0.92). The phase two recommendation reduced deaths caused by HNSCC (HR^e^: 0.86, 95% CI, 0.66–0.93).

### Treatment heterogeneity

**Figure 4:**
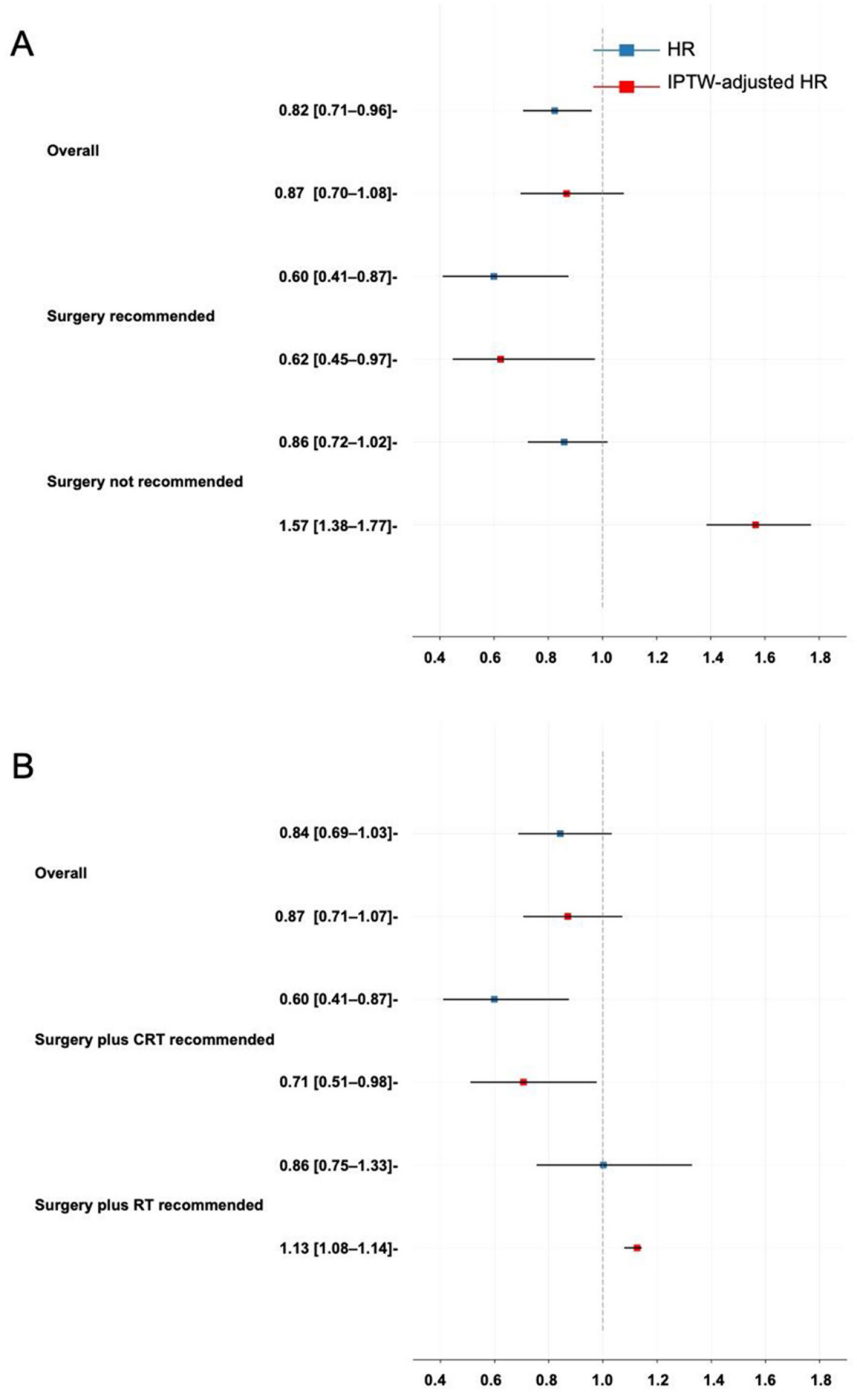
Treatment heterogeneity. **(A)** Treatment heterogeneity in the phase one recommendation; **(B)** Treatment heterogeneity in the phase two recommendation. HR, hazard ratio; IPTW, inverse probability treatment weighting.

Treatment heterogeneity can be captured by the presence of varied average treatment effects (ATEs) across different subgroups, indicating that patients with different characteristics respond heterogeneously to the same treatment. The patients were divided into the surgery recommended (SR) and surgery not recommended (SNR) groups based on the ITE that BITES predicted in the phase one recommendation. Similarly, the surgery plus CRT recommended (SCR) and surgery plus RT recommended (SRR) groups were established. The HR and HR^e^ were calculated to visualize the ATE in the overall patients and those subgroups. IPTW was used to adjust for tumor size, tumor locations, laterality, TNM stages, and demographic features. These results are presented in Figures 4A, B for the phase one and phase two recommendations, respectively.

In CRT vs. surgery plus RT/CRT, the ATE reflected the protective effect of surgery compared with CRT. Surgery demonstrated a very weak and statistically insignificant protective effect in all patients (HR^e^: 0.87, 95% CI, 0.70–1.08). However, it showed a protective effect in the SR group (HR^e^: 0.60, 95% CI, 0.45–0.97) and a risky effect in the SNR group (HR^e^: 1.57, 95% CI, 1.38–1.77).

The ATE of surgery plus CRT compared with surgery plus RT was not statistically significant in all patients (HR^e^: 0.87, 95% CI, 0.71–1.07). It became favorable in the SCR group (HR^e^: 0.71, 95% CI, 0.51–0.98) and not favorable in the SRR group (HR^e^: 1.13, 95% CI, 1.08–1.14).

### Therapeutic insights and model interpretation

**Figure 5:**
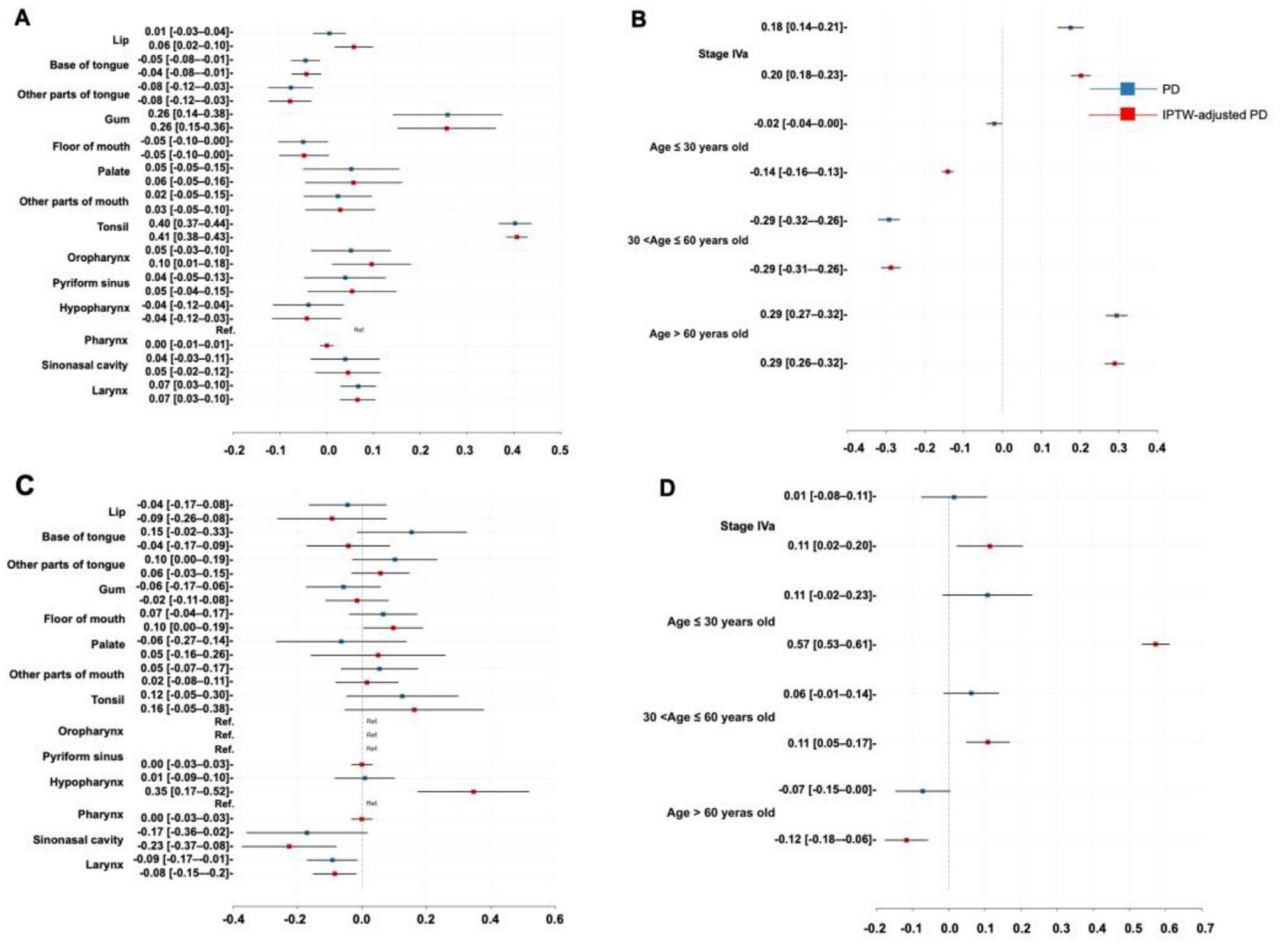
Therapeutic insights. **(A)** Probability difference regarding tumor location in the phase one recommendation; **(B)** Probability difference regarding age and TNM stage in the phase one recommendation; **(C)** Probability difference regarding tumor location in the phase two recommendation; **(D)** Probability difference regarding age and TNM stage in the phase two recommendation. PD, probability difference; IPTW, inverse probability treatment weighting.

Here, the PD and IPTW-adjusted PD (PD^e^) were used to quantify the impact of tumor location, age, and TNM stage on treatment selection. Figures 5A, B represent the probability differences for the phase one recommendation, while Figures 5C, D show similar results for the phase two recommendation. The PD represented the probability that a patient with the characteristic was recommended for surgery and surgery plus CRT minus the probability in the absence of the characteristic in phase one and phase two, respectively, whereas the IPTW correction provided a more unbiased result.

For the phase one recommendation, a higher likelihood of being recommended to receive surgery was found in the patients with tumors in the tonsil (PD^e^: 40.60%, 95% CI: 38.30%−42.90%), lip (PD^e^: 5.78%, 95% CI: 1.65%−9.90%), gum (PD^e^: 25.60%, 95% CI: 15.10%−36.10%), oropharynx (PD^e^: 9.57%, 95% CI: 1.13%−18.00%), and larynx (PD^e^: 6.57%, 95% CI: 2.78%−10.40%) subsites, those with stage IVa (PD^e^: 20.26%, 95% CI: 17.67%−22.85%), and those older than 60 years of age (PD^e^: 29.00%, 95% CI: 26.40%−31.50%), with specific likelihood listed accordingly in the PD^e^ values. In contrast, the patients with tumors located at the base of the tongue (PD^e^: −4.37%, 95% CI: −7.52%−1.21%), other parts of the tongue (PD^e^: −7.86%, 95% CI: −12.43%−3.29%), and those aged 30 to 60 years (PD^e^: −28.74%, 95% CI: −31.27%−26.21%) were less likely to be recommended for surgery.

For the phase two recommendation, factors such as floor of mouth carcinoma (PD^e^: 9.68%, 95% CI: 0.40%−19.00%), hypopharyngeal carcinoma (PD^e^: 34.6%, 95% CI: 17.20%−51.90%), stage IVa (PD^e^: 11.34%, 95% CI: 2.17%−20.50%), age between 30 and 60 years (PD^e^: 10.80%, 95% CI: 4.78%−16.90%), and age under 30 years (PD^e^: 57.20%, 95% CI: 53.40%−61.10%) were associated with a greater likelihood of being recommended for surgery plus CRT. On the other hand, surgery plus RT was more likely to be recommended for the patients with sinonasal cancer (PD^e^: −22.60%, 95% CI: −37.32%–−7.91%), laryngeal cancer (PD^e^: −8.46%, 95% CI: −15.20%–−1.74%), and those older than 60 years (PD^e^: −11.70%, 95% CI: −17.70%–−5.74%).

**Figure 6:**
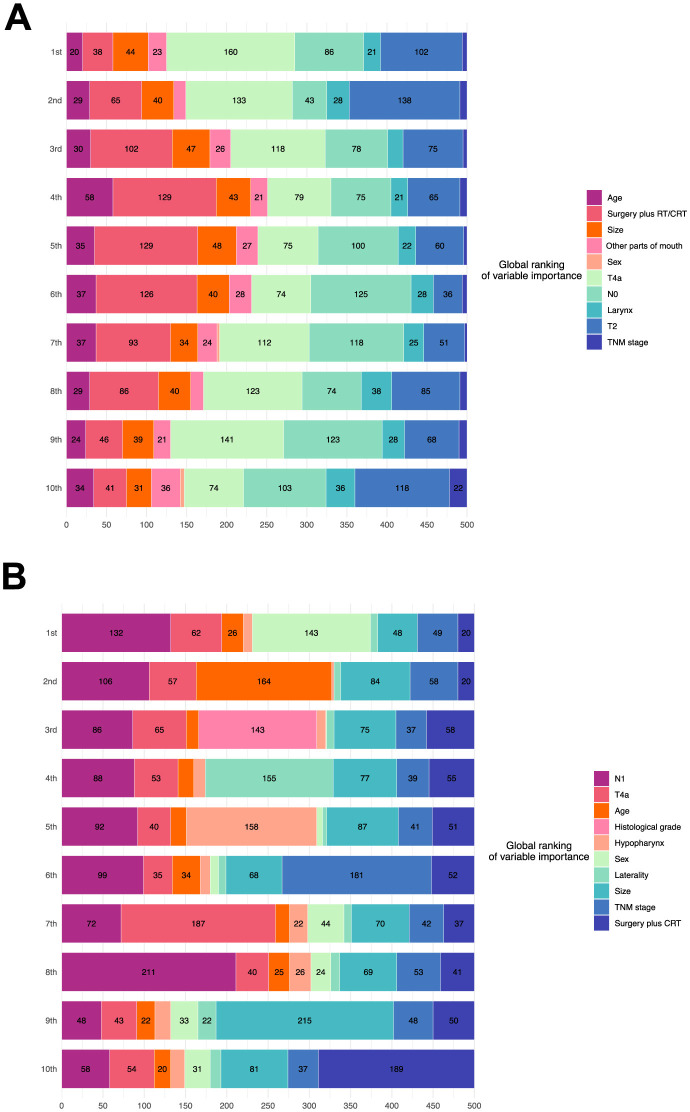
Model interpretation based on SurvSHAP(t). **(A)** Interpretation of the model of the phase one recommendation. **(B)** Interpretation of the model of the phase two recommendation. RT, radiation; CRT, chemoradiation.

Figures 6A, B visualize the eight most important variables, sorted by the aggregated Shapley values, for the overall model outputs for the phase one and phase two recommendations using SurvSHAP(t). These results were calculated over 500 random observations in the testing set. The horizontal bars represent the number of observations for which the importance of the variable, represented by a given color, was ranked as first, second, and so on.

According to the phase one model, advanced T stage was the most important feature, followed by N stage, age, and treatment. N stage, age, and histological grade significantly affected the outputs of the phase two model.

## Discussion

Surgery plus adjuvant RT is the classic therapy for patients with locally advanced HNSCC (3), while the use of adjuvant CRT has become increasingly popular (4). In terms of organ preservation, patients with advanced T stage or multiple lymph node involvement have been found to benefit from CRT (2). However, the treatment guidelines are still primarily population-based, and considering treatment heterogeneity, the optimal treatment plan for a patient needs to be considered at the individual level (8).

In this study, we developed and compared several models to provide individualized treatment recommendations for patients with locally advanced HNSCC. After thorough validation and bias control, BITES, a deep learning-based approach, demonstrated the best performance, prolonging patient survival by 4 to 10 months over 5 years. It outperformed real-world physician choices, widely used models, and NCCN guidelines, showcasing its potential to improve clinical treatment decisions by addressing complex treatment heterogeneity and non-linear interactions more effectively than traditional models such as CPH and RSF (29, 30).

We believe the advantage of BITES lies in its superior feature extraction capability and its representation-based causal inference method#. Its deep learning framework captures complex non-linear relationships, surpassing the limitations of traditional models such as CPH, which relies on constant hazard ratio assumptions, and RSF, which struggles with high-dimensional data (30). Through representation learning, it effectively balances covariates between treatment groups, reducing bias and improving ITE estimation (29), while traditional models are largely affected by selection bias in observational data#. In addition, BITES directly optimizes for the ITE, providing more precise treatment recommendations compared to DeepSurv, which focuses primarily on survival risk prediction (31). The shared and risk network architecture of BITES further enhances interpretability, making it particularly well-suited for clinical applications (29). These strengths position BITES as the most effective model for personalized treatment recommendations in this study and make it more suitable for individualized causal inference tasks.

Our quantitative results are consistent with the majority of the literature. In the phase one recommendation, we found that the patients older than 60 years were 29% more likely to be recommended for surgery than the remaining patients, which is supported by studies (32) indicating that the efficacy of chemotherapy decreases with the increasing age of the patient. Similar results were found in the patients with onset sites in the lip (33), gum (34), oropharynx (35), larynx (36), and tonsil (37), as well as in those with stage Iva (38). In addition, Foster et al. (39) found lower rates of osteonecrosis in tongue cancer patients treated with CRT, supporting the greater likelihood of them being recommended for CRT.

In the phase two recommendation, surgery plus RT was more frequently recommended for the older patients due to the reduced efficacy of chemotherapy (40). In addition, the better efficacy of this approach has been proven in patients with sinonasal cancer (41) and laryngeal cancer(36). Conversely, patients with stage Iva (42), onset sites in the hypopharynx (43), and floor of the mouth (44, 45) are found to benefit more from adjuvant CRT.

Maximizing patient survival and providing a satisfactory quality of life are priorities for physicians. Compared to conventional guidelines, DL models can not only personalize treatment but also quantify the benefits of each treatment and provide a visual platform for doctors and patients to communicate with each other. With the continuous improvement of DL models, the application can be extended to other areas, such as risk identification and imaging prediction, simplifying clinical diagnosis and treatment.

### Limitations

The complete inclusion of variables and diverse outcomes is still an area of improvement. The SEER database lacks some important clinical variables, such as human papillomavirus status and vascular invasion, hindering more accurate modeling. In addition, other survival outcomes are also important considerations for patients when choosing a treatment plan, whereas our model solely focused on whether to perform organ preservation.

## Conclusion

In this study, we developed a personalized treatment recommendation system for patients with locally advanced HNSCC using DL models. BITES demonstrated the ability to identify patients who can achieve organ preservation with CRT and to guide maximum survival. Comprehensive clinical data and further refinement of DL models can enable more accurate predictions in the future, ultimately achieving the potential of precision medicine.

## Data Availability

The datasets used in this study are available in online repositories. The original data can be accessed through the Surveillance, Epidemiology, and End Results (SEER) database at https://seer.cancer.gov/data/.
