# Supplementary material for: Individualized treatment recommendations for patients with locally advanced head and neck squamous cell carcinoma utilizing deep learning
Source: Front Med (Lausanne). 2025 Jan 6;11:1478842. doi: 10.3389/fmed.2024.1478842 (PMC11744519; doi:10.3389/fmed.2024.1478842)
Supplement: Supplementary file 1 [file Table_1.docx]

Table S1: Model's protection against each cause of death

| chemoradiation versus surgery plus radiation/chemoradiation | | |
| --- | --- | --- |
|  | HR | IPTW-adjusted HR |
| Head and neck cancer | 0.76 (0.65–0.90) | 0.84 (0.69­–0.94) |
| Miscellaneous malignant cancer | 0.83 (0.65–1.08) | 0.82 (0.53–1.27) |
| Lung diseases | 0.80 (0.49–1.30) | 0.94 (0.48–1.84) |
| Cardiovascular diseases | 0.63 (0.46–0.85) | 0.66 (0.45–0.96) |
| Infectious diseases | 0.78 (0.42–1.47) | 0.62 (0.22–1.74) |
| Adverse effect | 0.52 (0.27­–0.92) | 0.68 (0.38–0.92) |
| Surgery plus radiation versus surgery plus chemoradiation | | |
|  | HR | IPTW-adjusted HR |
| Head and neck cancer | 0.85 (0.65–0.91) | 0.86 (0.66–0.93) |
| Miscellaneous malignant cancer | 1.02 (0.67–1.56) | 0.91 (0.60–1.37) |
| Lung diseases | 0.71 (0.29–1.71) | 0.61 (0.27–1.39) |
| Cardiovascular diseases | 0.93 (0.53–1.63) | 0.64 (0.36–1.16) |
| Infectious diseases | 0.74 (0.25–2.19) | 0.55 (0.16–1.90) |
| Adverse effect | 1.73 (0.59­–5.06) | 1.66 (0.57–4.83) |

HR, hazard ratio with competing risk; IPTW, inverse probability treatment weighting; IPTW-adjusted HR with competing risk was calculated using marginal structural Cox proportional hazard model.
